# Supplementary material for: Multiple symmetry protected BIC lines in two dimensional synthetic parameter space
Source: Nanophotonics. 2023 Feb 6;12(13):2405–13. doi: 10.1515/nanoph-2022-0781 (PMC11501203; doi:10.1515/nanoph-2022-0781)
Supplement: Supplementary file 1 — Supplementary Material Details [file j_nanoph-2022-0781_suppl.docx]

Supplementary Material

To demonstrate the existence of multiple BIC modes and their properties in the lossy regime, we first calculated the eigenfrequency variations of nanohole metasurfaces with varied asymmetric parameters p and q respectively with considering metal loss, as depicted in Figure S1a, b. The variation of the eigenfrequencies of the four modes is consistent with that in the lossless regime. Figure S1c shows the Ex distribution of four modes. Three BIC modes X1, X2, Y1 and N1 mode are indicated by red and green circles, respectively.


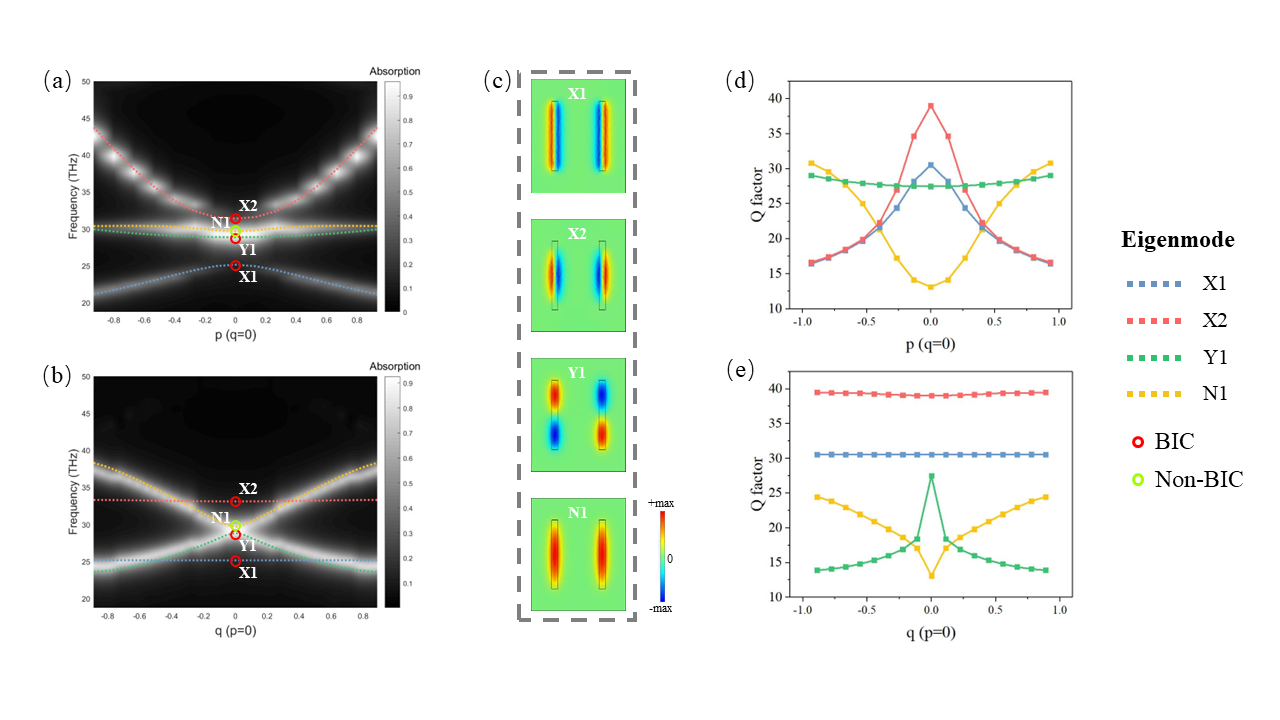


Figure S1. Multiple BIC modes investigation in 1D parameter space with considering the metal loss. (a) Simulated eigenfrequency variations of four modes X1, X2, Y1, N1 and corresponding absorption spectra variations with varied asymmetry parameter p while q fixed as 0. The position of the BICs (non-BIC) are marked by red (green) circles. (b) Simulated eigenfrequency variations of four modes and corresponding absorption spectra variations with varied asymmetry parameter q while p fixed as 0. (c) The Ex distribution of four modes at high-order symmetry point (p=0, q=0). Variations of Q factors with varied asymmetry parameter p (q=0) (d) and asymmetry parameter q (p=0) (e) respectively for four modes.

Then, we calculate the absorption spectra of designed metasurfaces as a function of the asymmetric parameters p and q, respectively, as shown in Figure S1a, b. The absorption spectra of each mode in lossy system show the same variation tendency as that in the lossless system, except for the broaden linewidth of absorption peaks due to metal loss. we also calculated the Q factor variations of these modes as the asymmetric parameters p or q change, as shown in Figure S1d, e. We can see that the results are also consistent with that in the lossless system, except for the decreased Q factors due to metal loss.

The calculated Q factors and real part of eigenfrequency of four modes X1, X2, Y1, and N1 in p-q space with considering the metal loss are shown in Figure S2 and Figure S3, respectively. Due to the metal loss, the Q factors of BICs are decreased but vary with same tendency as that in the lossless system. As for the real part of eigenfrequency, with varied asymmetry parameters p and q, four modes exhibit different characteristics. Thus, by modulating the asymmetry parameters in p-q space, both in the lossless and lossy system, proposed nanohole metasurface array design can achieve flexibly optical properties tuning of BICs.


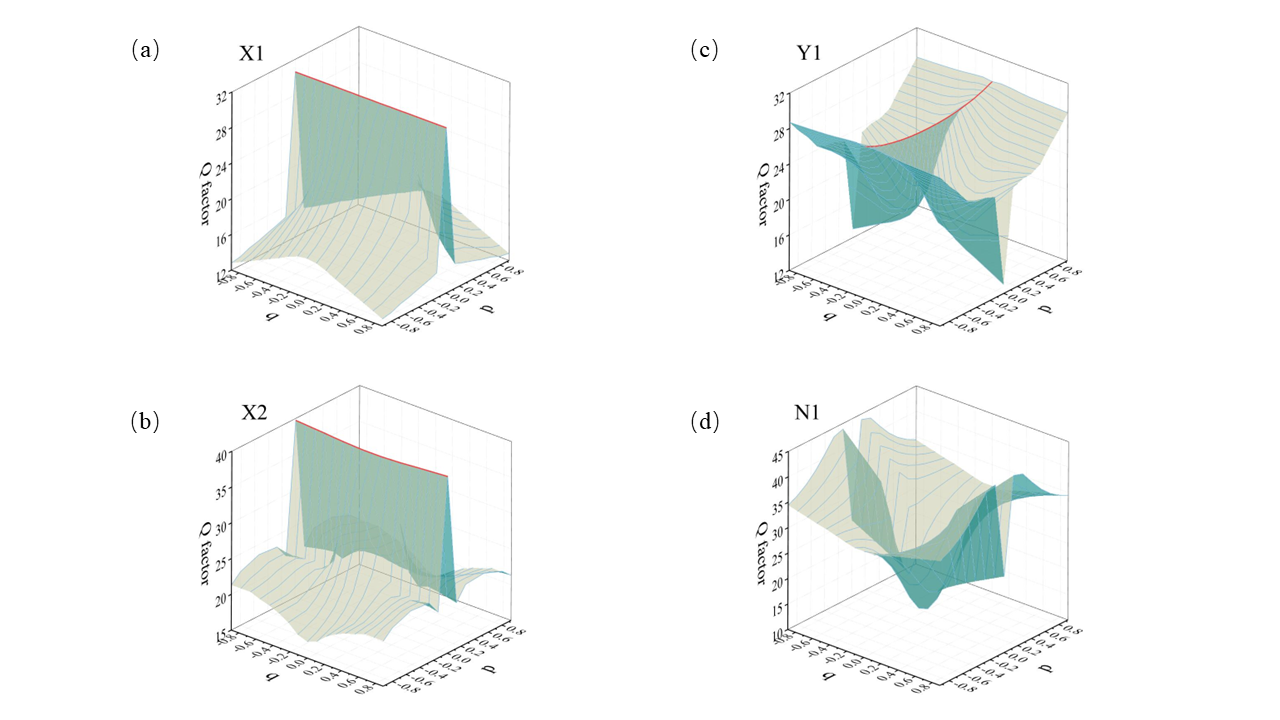


Figure S2. Multiple BIC modes investigation in synthetic p-q space with considering the metal loss. Distribution of Q factor in p-q space for (a) X1, (b) X2, (c) Y1 and (d) N1 modes. BIC lines are marked by red lines.


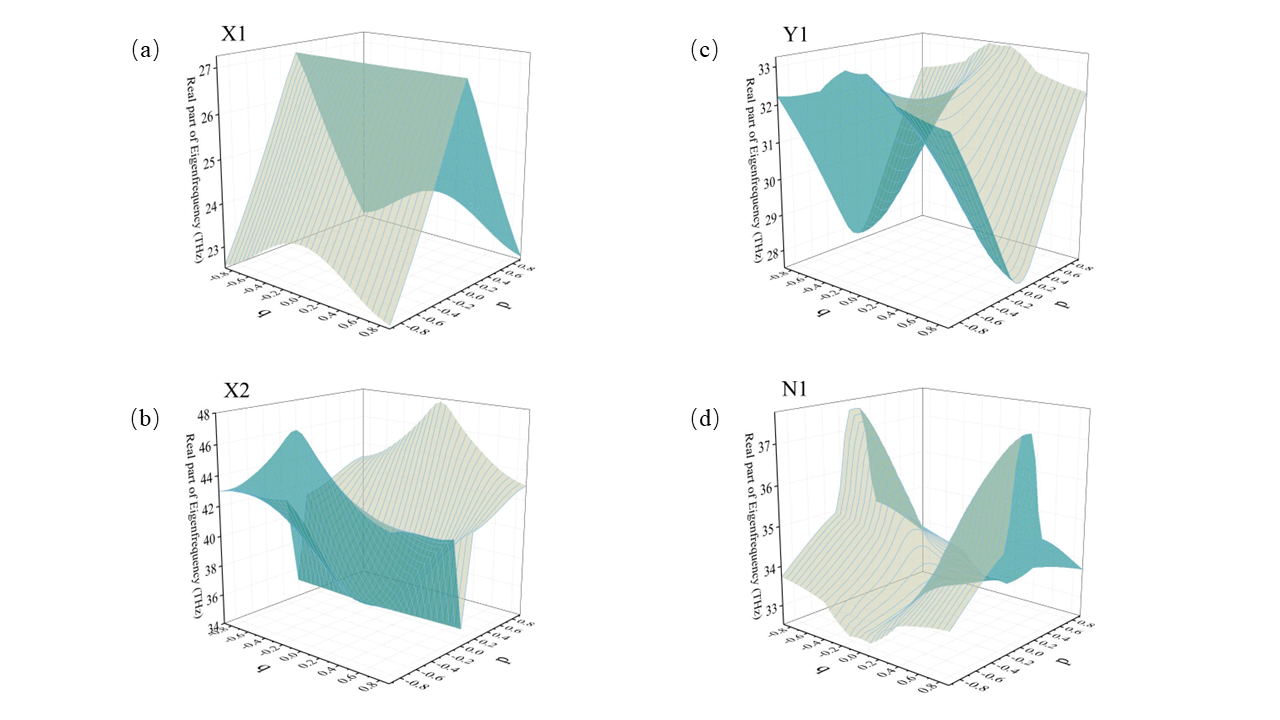


Figure S3. Multiple BIC modes investigation in synthetic p-q space with considering the metal loss. Distribution of the real part of eigenfrequency in p-q space for (a) X1, (b) X2, (c) Y1 and (d) N1 modes.

For further verify these modes (X1, X2 and Y1) under symmetry protection are BICs, we analyzed the out-of-plane electric field distribution of the discussed modes. The electric field Ex and |E| distributions in the x-z plane (y=0 cross section) for each of the four modes at four points in p-q space, are shown in Figure S4 and Figure S5, respectively. The BIC modes are marked by red dashed box. It can be seen that the field distributions of these BIC modes are all localized inside the structure with no outward radiation. When p or q changes, the electric field distribution appears outside the structure, which means that the quasi-BIC modes start to radiate outward. In contrast, for non-BIC mode N1, outward radiation always exists at all points. Therefore, the above results can prove that modes X1, X2 and Y1 under symmetry protection are BICs.


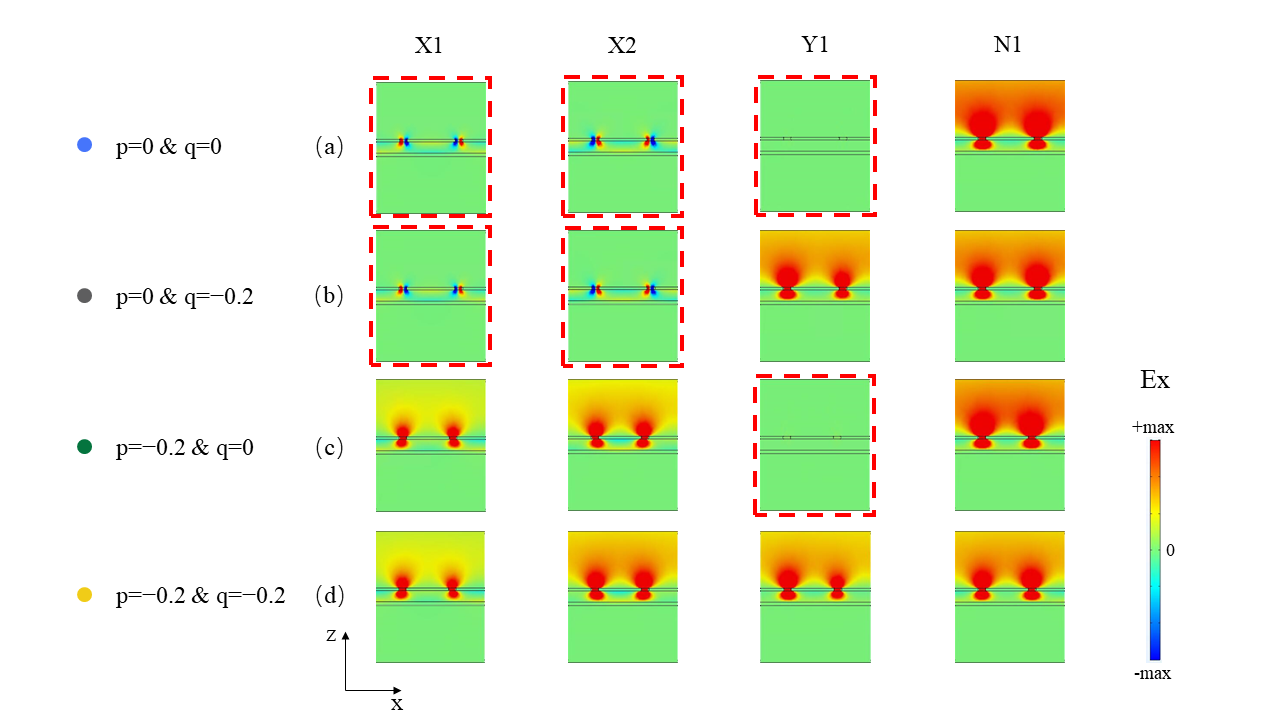


Figure S4. Electric field distribution of four modes in p-q space. The out-of-plane Ex distribution for X1, X2, Y1 and N1 modes at four picked points where p=0, q=0 (a), p=0, q=-0.2 (b), p=-0.2, q=0 (c) and p=-0.2, q=-0.2 (d). The red dashed box represents the BIC modes.


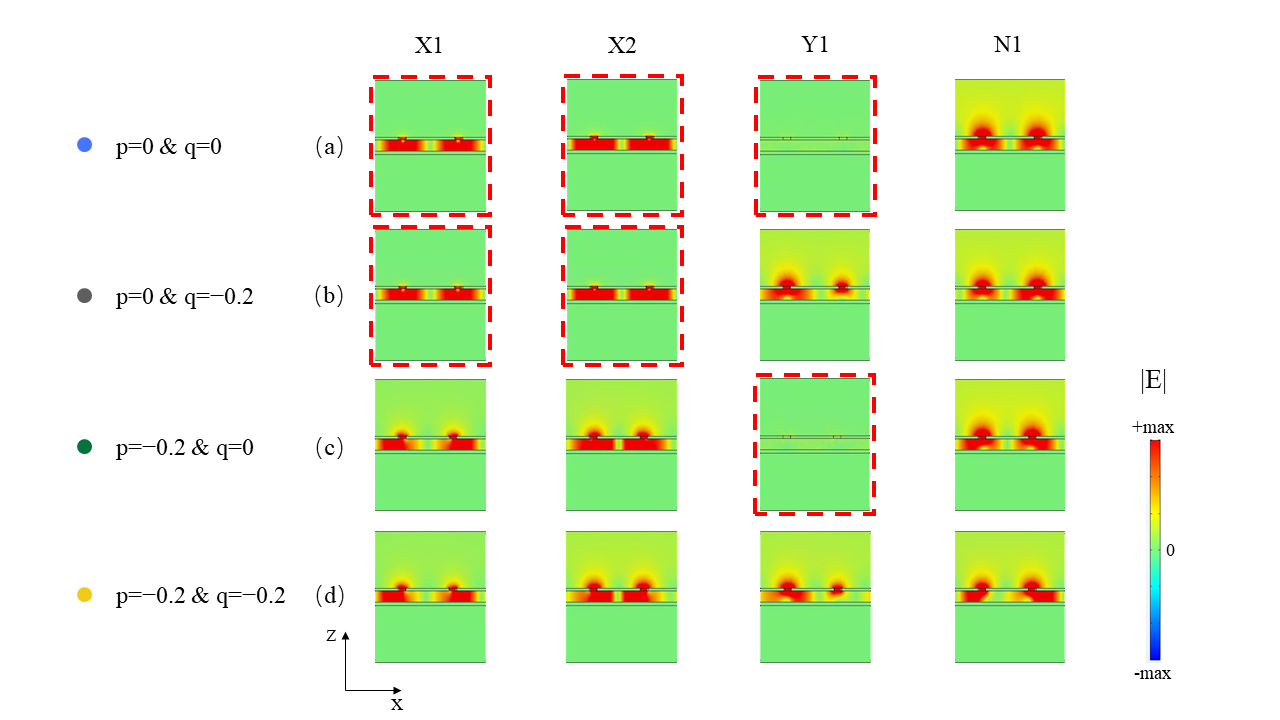


Figure S5. Electric field distribution of four modes in p-q space. The out-of-plane |E| distribution for X1, X2, Y1 and N1 modes at four picked points where p=0, q=0 (a), p=0, q=-0.2 (b), p=-0.2, q=0 (c) and p=-0.2, q=-0.2 (d). The red dashed box represents the BIC modes.
